# Corneal confocal microscopy may help to distinguish Multiple System Atrophy from Parkinson’s disease

**DOI:** 10.1038/s41531-024-00680-8

**Published:** 2024-03-16

**Authors:** Xuebin Niu, Peixiao Yin, Chenyang Guan, Qiuyue Shao, Guiyun Cui, Kun Zan, Chuanying Xu

**Affiliations:** 1 Department of Neurology, The Affiliated Hospital of Xuzhou Medical University, Xuzhou, Jiangsu China; 2 Department of Neurology, The First Clinical College, Xuzhou Medical University, Xuzhou, Jiangsu China

**Keywords:** Parkinson's disease, Neurodegeneration

## Abstract

Multiple system atrophy (MSA) and Parkinson’s disease (PD) have clinical overlapping symptoms, which makes differential diagnosis difficult. Our research aimed to distinguish MSA from PD using corneal confocal microscopy (CCM), a noninvasive and objective test. The study included 63 PD patients, 30 MSA patients, and 31 healthy controls (HC). When recruiting PD and MSA, questionnaires were conducted on motor and non-motor functions, such as autonomic and cognitive functions. Participants underwent CCM to quantify the corneal nerve fibers. Corneal nerve fiber density (CNFD) and corneal nerve fiber length (CNFL) values in MSA are lower than PD (MSA vs. PD: CNFD, 20.68 ± 6.70 vs. 24.64 ± 6.43 no./mm^2^, *p* < 0.05; CNFL, 12.01 ± 3.25 vs. 14.17 ± 3.52 no./mm^2^, *p* < 0.05). In MSA + PD (combined), there is a negative correlation between CNFD and the Orthostatic Grading Scale (OGS) (r = −0.284, *p* = 0.007). Similarly, CNFD in the only MSA group was negatively correlated with the Unified Multiple System Atrophy Rating Scale I and II (r = −0.391, *p* = 0.044; r = −0.382, *p* = 0.049). CNFD and CNFL were inversely associated with MSA (CNFD: β = −0.071; *OR*, 0.932; 95% CI, 0.872 ~ 0.996; *p* = 0.038; CNFL: β = −0.135; *OR*, 0.874; 95% CI, 0.768–0.994; *p* = 0.040). Furthermore, we found the area under the receiver operating characteristic curve (ROC) of CNFL was the largest, 72.01%. The CCM could be an objective and sensitive biomarker to distinguish MSA from PD. It visually reflects a more severe degeneration in MSA compared to PD.

## Introduction

Multiple system atrophy (MSA) and Parkinson’s disease (PD) are common neurodegenerative diseases classified as α-synucleinopathies^1^, sharing similar clinical features, such as autonomic dysfunction and parkinsonism^2^. Autopsy studies conducted on individuals with MSA and PD have revealed an approximate misdiagnosis rate of 20%^3^, indicating a challenge in differentiating between the two disorders in the early stage. Furthermore, this high misdiagnosis rate and rapid progression of MSA in patients have significant negative impacts on the treatment and quality of life of individuals with MSA. Therefore, a novel promising biomarker able to differentiate between these two diseases is urgently needed.

In recent years, skin biopsy in many studies was used as an emerging biomarker to detect the deposits of α-synuclein to identify synucleinopathies^4,5^, and it is well developed in the identification of MSA and PD^6,7^. However, it is an invasive procedure that requires expertize and may cause pain and discomfort to the patient. In addition, there are studies using imaging techniques to examine the brain’s functional structure to differentiate the two disorders. The drug’s effect on brain function is unknown, so the technology is still inadequately studied^8,9^. Confocal corneal microscopy (CCM) is a rapid imaging technique for the noninvasive visualization and quantification of corneal nerve fibers^10^. Corneal nerves, originating from the ophthalmic branch of the trigeminal nerve, are considered a part of the peripheral nervous system. Recently, CCM has been increasingly recognized as a valuable tool for identifying PD with more rapid motor progression and varying degrees of non-motor symptoms^10–12^. Small fiber neuropathy (SFN) is a form of peripheral neuropathy that can impact both autonomic and somatosensory nerves. Several studies have suggested the presence of autonomic and somatosensory dysfunction in MSA^7,13^, and in MSA, abnormal aggregation of α-synuclein primarily occurs in somatosensory fibers^14^. However, there is currently limited research by CCM in MSA, and no large sample studies are using CCM to differentiate PD from MSA.

In this study, CCM was used to quantify the corneal nerve fibers of PD, MSA patients, and healthy controls, to observe differences in corneal parameters among them, and to evaluate whether CCM could sensitively distinguish PD and MSA.

## Results

### **Clinical characteristics and CCM parameters**

63 PD, 30 MSA, and 31 HC underwent ophthalmic evaluation and were included in the study (15 PD patients with diabetes and 2 MSA patients with diabetes were excluded). We compared clinical characteristics and CCM parameters among HC, PD, and MSA patients. The Mann-Whitney U test revealed that PD showed longer disease duration than MSA (4.50 ± 6.00 years vs. 3.00 ± 2.00 years, *p* < 0.001). Moreover, there were no significant differences in the age between PD and MSA (Table 1).

**Table 1:** Demographic and clinical characteristics of participants

	Controls(31)	PD(63)	MSA(30)
Sex(M/F)	13/18	26/37	14/16
Age, years	60.23 ± 10.73^a***^	66.43 ± 6.92	63.80 ± 8.00
Disease duration, years	NA	4.50 (2.00,8.00)^b***^	3.00 (2.00,4.00)
H-Y stage	NA	2.00 (1.50,2.50)^b***^	3.00 (2.50,3.00)
UPDRS-III	NA	29.84 ± 16.09^b***^	42.73 ± 22.11
Moca	NA	19.62 ± 6.37^b***^	14.00 ± 6.90
SCOPA	NA	16.63 ± 10.54^b***^	30.97 ± 11.83
NMSS	NA	39.00 (22.00,74.00)^b^***	64.50 (44.00,140.00)
OGS	NA	1.76 ± 3.53^b***^	7.10 ± 6.50
UMSARS-I	NA	NA	18.40 ± 10.22
UMSARS-II	NA	NA	21.37 ± 10.31
CNFD no./mm^2^	31.36 ± 7.39^a***^	24.64 ± 6.43^b***^	20.68 ± 6.70^c***^
CNBD no./mm^2^	39.69 ± 15.01	33.06 ± 23.90	24.12 ± 14.66^c***^
CNFL mm/mm^2^	16.69 ± 3.40^a***^	14.17 ± 3.52^b***^	12.01 ± 3.25^c***^

Numbers are expressed as mean ± SD or median (interquartile range). The normality of the distribution was evaluated using the Shapiro-Wilk test. To compare multiple groups, an analysis of variance was conducted with Bonferroni as the post hoc test. The independent-sample t-test was employed to compare means of data that follow a normal distribution and the Mann-Whitney U test was employed for nonparametric data. Chi-square tests and Fisher’s exact tests were used to compare categorical variables.

*NA* not available, *UPDRS-III* unified Parkinson’s disease rating scale 3, *SCOPA-AUT* the scale for outcomes in PD for autonomic symptoms, *UMSARS-I and UMSARS-II* the Unified Multiple System Atrophy Rating Scale I and II, *OGS* the Orthostatic Grading Scale, *Moca* the Montreal Cognitive Assessment scale, *NMSS* the NonMotor Symptoms Scale, *CNFD* corneal nerve fiber density, *CNBD* corneal nerve branch density, *CNFL* corneal nerve fiber length.

***P < 0.001.

^a^ Differences between PD and Controls.

^b^ Differences between PD and MSA.

^c^ Differences between MSA and Controls.

### Corneal nerve fiber parameters

**Fig. 1:**
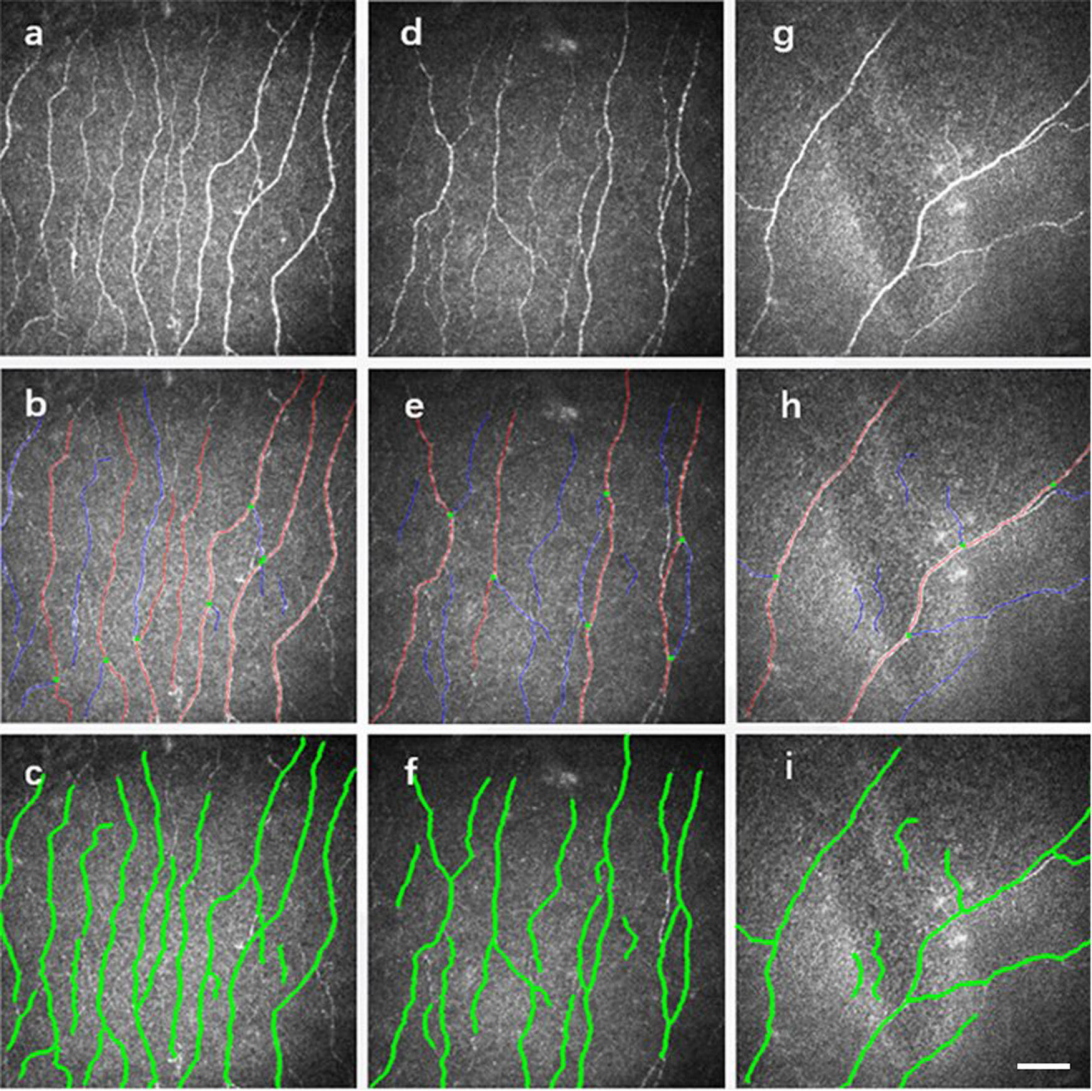
High-quality and representative corneal confocal microscopy images. Panels **a**, **d**, **g** represent the original CCM images of HC, PD, and MSA; Figures **b**, **e**, **h** and **c**, **f**, **i** were analyzed using the automated version (ACCMetrics) to represent the detected nerves and the marked images between the groups respectively. *PD* Parkinson’s disease, *MSA* Multiple system atrophy.

**Fig. 2:**
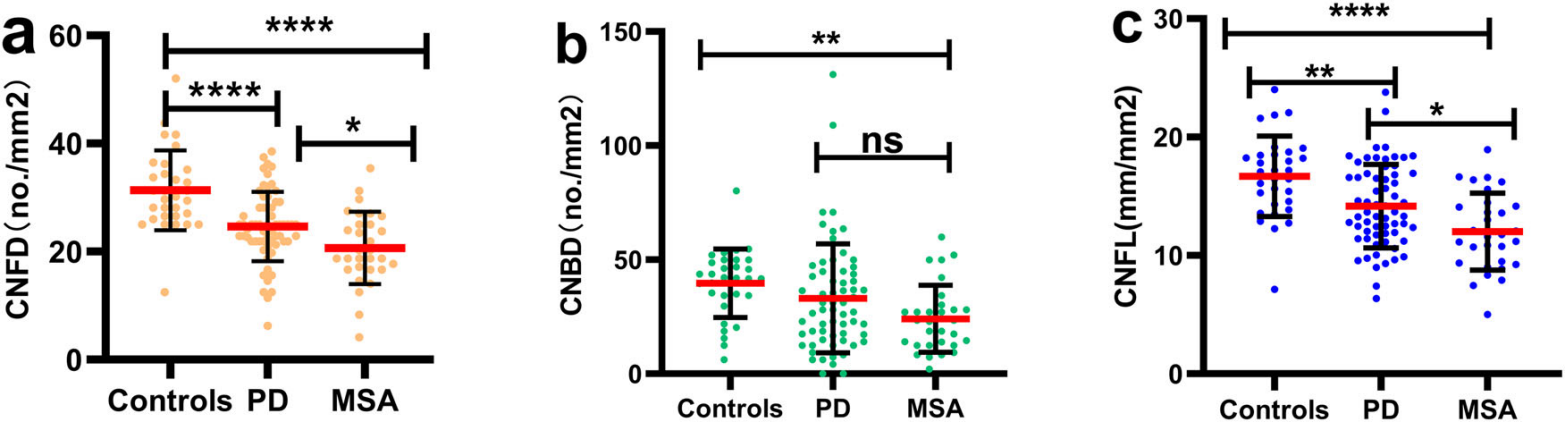
CCM parameters between groups. **a**–**c** indicate the differences in CNFD, CNBD and CNFL values among the three groups. The differences in CNFD and CNFL values among the HC, PD, and MSA groups are significant. Errors bars represent mean ± standard deviation (*p < 0.5; **p < 0.01; ***p < 0.001; ****p < 0.0001; ns non-significant). CNFD corneal nerve fiber density, CNBD corneal nerve branch density, CNFL corneal nerve fiber length.

Representative CCM images of the HC, PD, and MSA groups are presented in Fig. 1. The MSA group significantly reduced nerve fiber density compared with the other two groups. The differences in CNFD, CNBD, and CNFL values among the HC, PD, and MSA groups are shown in Fig. 2. The MSA group had significantly lower CNFD and CNFL values than the PD groups (MSA vs. PD: CNFD, 20.68 ± 6.70 vs. 24.64 ± 6.43 no./mm^2^, *p* < 0.05; CNFL, 12.01 ± 3.25 vs. 14.17 ± 3.52 no./mm^2^, *p* < 0.05).

### The correlations between CCM parameters and clinical scores

**Fig. 3:**
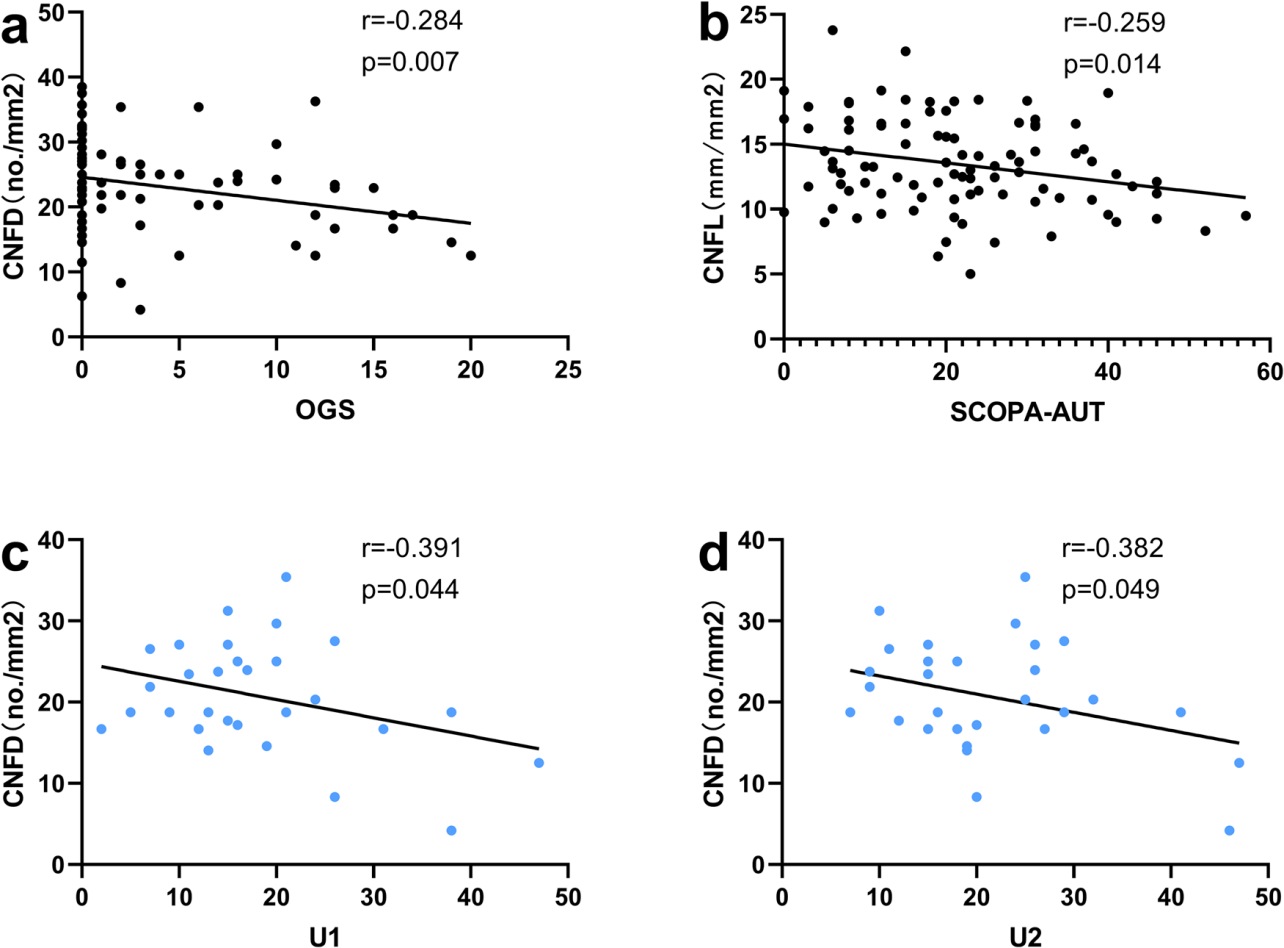
The correlations between CCM parameters and clinical scores. Partial correlation analysis was utilized to examine the correlation between CCM parameters and clinical measures. The correlation analyses were performed in MSA + PD (combined) with regard to the OGS (**a**) and SCOPA-AUT (**b**), and only in MSA with regard to UMSARS (**c, d**). CNFD corneal nerve fiber density, CNFL corneal nerve fiber length, SCOPA-AUT the scale for outcomes in PD for autonomic symptoms, UMSARS-I and UMSARS-II the Unified Multiple System Atrophy Rating Scale I and II, OGS the Orthostatic Grading Scale.

Correlations between CCM parameters and clinical scales were examined in the MSA group and the PD group (Fig. 3). After adjusting for age, sex, and disease duration, a negative correlation was observed between CNFL and the SCOPA-AUT scores (*r* = −0.259, *p* = 0.014), which were performed in MSA + PD (combined). Additionally, a negative correlation was found between CNFD and the OGS (*r* = −0.284, *p* = 0.007) in MSA + PD (combined). However, the correlation between OGS and SCOPA-AUT and CCM parameters was not significant when analyzing MSA and PD as separate groups. In the MSA group, CNFD negatively correlated with the UMSARS scores for domains I and II (*r* = −0.391, *p* = 0.044; *r* = −0.382, *p* = 0.049).

### The association between CCM parameters and MSA

We controlled for confounders and performed binary logistic regression analysis to determine whether CCM parameters have a relationship with MSA. We found that CNFD and CNFL negatively associated with MSA (CNFD: *β* = −0.071; *OR*, 0.932; 95% CI, 0.872–0.996; *p* = 0.038; CNFL: *β* = −0.135; *OR*, 0.874; 95% CI, 0.768–0.994; *p* = 0.040) (Table 2).

**Table 2:** The association between CCM parameters and MSA

	*β*	OR(95% CI)	*P*
CNFD	−0.071	0.932 (0.872 to 0.996)	0.038
CNBD	−0.022	0.978 (0.953 to 1.004)	0.101
CNFL	−0.135	0.874 (0.768 to 0.994)	0.040

To mitigate the influence of confounding factors, age, sex, and disease duration were selected as covariates for binary logistic regression. We performed three logistic regressions for each CCM metric (CNFD, CNBD, and CNFL are the dependent variables in each regression, respectively. *CNFD* corneal nerve fiber density, *CNBD* corneal nerve branch density, *CNFL* corneal nerve fiber length.

### Diagnostic accuracy

**Fig. 4:**
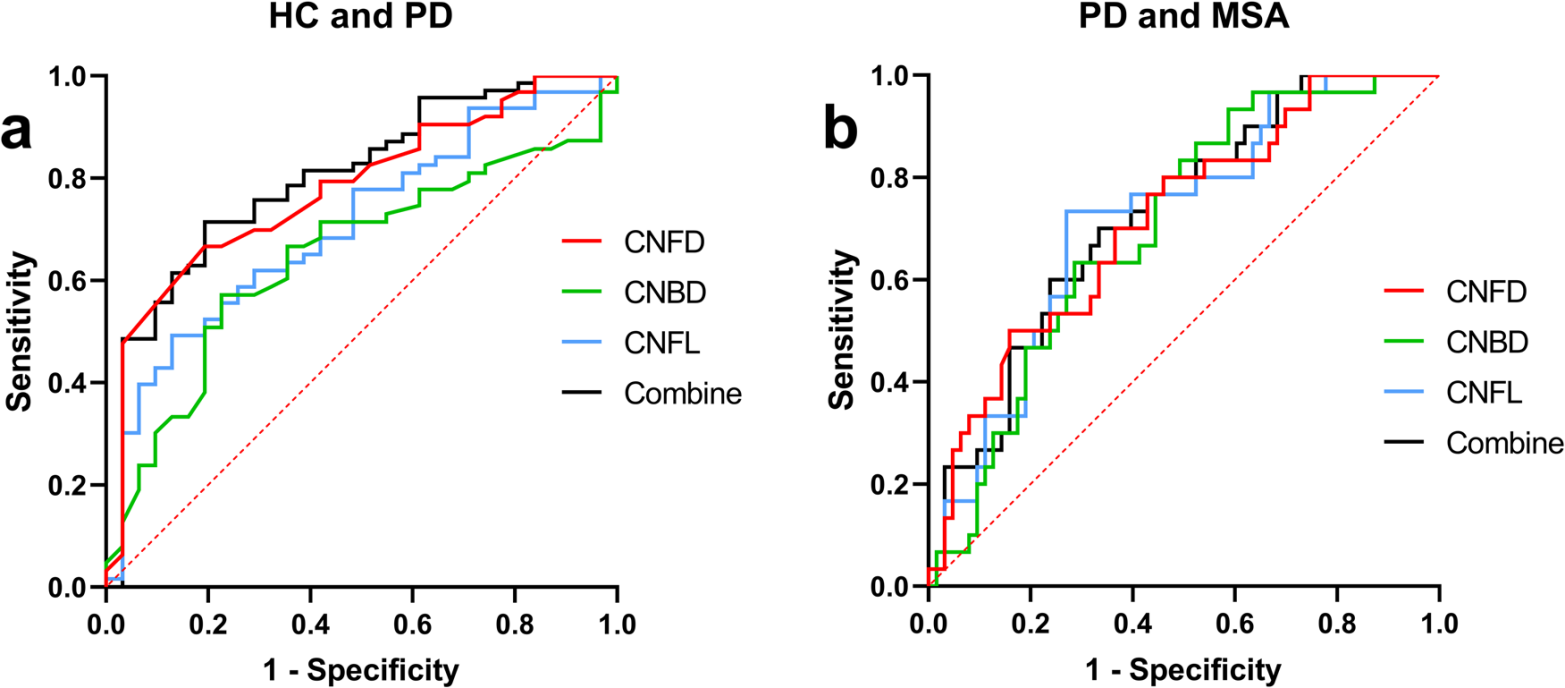
The ROC for CCM parameters to distinguish HC and MSA from PD. ROC was used to indicate the accuracy of CCM parameters in distinguishing HC (**a**) and MSA (**b**) from PD. After controlling for age, gender, and disease duration, the AUC value of CNFL in distinguishing MSA from PD is higher than other CCM parameters. CNFD corneal nerve fiber density, CNBD corneal nerve branch density, CNFL corneal nerve fiber length, these three parameters were used together.

After controlling for age, gender, and disease duration, the AUC value of CNFD in distinguishing MSA from PD was 71.72% (95% CI: 60.85–82.59%, sensitivity 50.00%, specificity 84.13%), and the AUC value of CNBD was 70.26%. (95% CI, 59.58–80.95%, sensitivity 63.33%, specificity 71.43%), CNFL was 72.01% (95% CI, 61.28–82.74%, sensitivity 73.33%, specificity 73.02%). When these three parameters were used together, the AUC increased by 72.33% (95% CI: 61.77–82.89%), the sensitivity was 70.00%, and the specificity was 66.6% (Fig. 4).

## Discussion

In this study, we utilized CCM technology to differentiate between MSA and PD objectively and sensitively. Our investigation revealed that MSA patients exhibited significantly reduced CNFD and CNFL compared to PD and HC. We also found a negative correlation between CNFL and SCOPA-AUT scale, as well as between CNFD and UMSARS. After accounting for confounding factors, we found CNFD and CNFL negatively associated with MSA. Furthermore, CNFL demonstrated high diagnostic accuracy in distinguishing MSA from PD.

Our study found that although MSA has a shorter course than PD, its clinical symptoms are more severe. This finding suggests that MSA may be a more rapidly progressing synucleinopathy compared to PD^15,16^ and may help to distinguish MSA from PD. However, relying solely on clinical symptoms for differential diagnosis poses significant challenges due to the subjective nature of clinical scales. Therefore, combining more objective indicators to ensure accurate disease diagnosis is crucial. Our study observed the values of CNFD and CNFL in MSA were lower than in PD, which aligns with the differences in clinical symptoms. Additionally, the findings showed the PD group had a lower CCM parameter than controls, consistent with previous studies^10,17^. CCM has been demonstrated as a rapid and objective technique for assessing corneal small nerve fiber damage^18^, and these significantly lower parameters compared to controls are also believed to be helpful in evaluating the rapid progression of the disease^10,19^. The study found that the CNBD in MSA patients was lower than controls. However, there was no significant difference in CNBD between patients with PD and controls. Previous studies have indicated that in the early stages of PD, there is a certain degree of increase in the number of corneal nerve fiber branches, suggesting the presence of a regeneration mechanism^12^. Therefore, it is possible that the manifestation of nerve regeneration in MSA patients is masked, possibly due to the characteristics of the disease or disruption of this compensatory mechanism. These findings suggest that CCM can be used as an objective indicator to distinguish between clinical presentations and that MSA may progress more rapidly than PD. Previous studies examining the differentiation of PD from other syndromes did not find any difference in corneal parameters, possibly due to small sample sizes^20,21^. In contrast, our study had a relatively large sample size, enhancing the interpretability of our data.

We found a correlation between corneal parameters, the autonomic nervous system scale, and the MSA scoring scale. This suggests that changes in corneal parameters are linked to impaired autonomic nervous system function and more significantly impairment of motor function in synucleinopathies. Previous research has demonstrated that a decrease in corneal parameters in PD is associated with a faster progression of motor symptoms and impairment of the autonomic nervous system, while there are fewer related studies in MSA. Therefore, our study establishes a connection between corneal parameters and their specific scales in MSA. Combined with our conclusion that MSA is more severe than PD based on UPDRS scores, the worsened CCM metrics indicate that MSA may specifically affect corneal nerves to make corneal nerve fiber damage more severe. However, it is important to note that further confirmation and clarification combined with pathological studies in corneal nerves are necessary. Furthermore, this further enhances the current understanding of the relationship between corneal nerve fibers and clinical assessment in central degenerative diseases. While MSA is primarily regarded as a disease of the central nervous system, recently emerging evidence suggests that peripheral nerve involvement becomes increasingly evident as the disease advances^22^. Our results indicate corneal nerve damage in MSA, thereby complementing existing evidence on the role of the peripheral nervous system in the early stages of MSA. Moreover, an association was found between corneal parameters and MSA. This association was particularly evident when the two indicators, CNFD and CNFL, were further reduced. Previous studies have shown that reductions in CNFD and CNFL are associated with severe peripheral neuropathy or neurodegeneration, and these two indicators are highly consistent^17,23,24^. Therefore, declines in these indicators suggest that small nerve fiber damage is more serious and may have a negative association with neurodegenerative diseases, especially MSA. In the future, it is necessary to combine pathological studies to provide a more comprehensive explanation for the observed decrease in corneal parameters in MSA and to conduct longitudinal follow-up studies to understand whether CCM could be a candidate biomarker to predict MSA progression.

MSA and PD are both α-Synuclein nucleoprotein diseases characterized by abnormal deposition of α-Synuclein nucleoprotein^15^. However, the deposition patterns differ between the two diseases. In MSA, abnormal a-synuclein accumulates mainly as glial cytoplasmic inclusions (GCIs) in oligodendrocytes, while in PD, a-synuclein deposits in Lewy bodies^25–27^. However, the pathological deposition of PD and MSA has not been demonstrated at the corneal level. Our results demonstrated a more severe reduction of corneal nerve parameters, suggestive of peripheral neurodegeneration, in MSA relative to PD and HC. Further studies are needed to understand whether this finding is reflected by corresponding abnormal a-syn deposits in these nerve fibers. Moreover, it would be valuable to include DLB patients and compare the differences in corneal nerve fibers among various synucleinopathies to reveal the possible mechanism.

ROC curve results in our study demonstrate that corneal parameters have a significant diagnostic utility in distinguishing between PD and MSA, particularly about the CNFL indicator. Many previous studies have found that CNFD is more stable and has better diagnostic performance^28,29^. Therefore, our study suggests that CNFL may be a more objective and sensitive indicator than CNFD, which can more accurately distinguish between PD and MSA.

This study has several limitations. Firstly, our study lacked autopsies of PD and MSA to confirm the diagnosis. Secondly, the cross-sectional nature of our study prevents us from establishing a causal relationship between corneal nerve fiber parameters and PD or MSA. Future longitudinal studies with larger sample sizes are needed to understand the underlying mechanisms further and validate our results. Thirdly, the quantification of corneal parameters is limited when using small area sizes. In future studies, it may be necessary to utilize wide-field imaging or include additional high-quality images to reduce potential bias. Moreover, it is essential to standardize CCM techniques and interpretation of results for better comparability across studies. Finally, we acknowledge that the evaluation of clinical scales is subjective. In our study, the MSA group, which consisted of younger individuals with shorter disease duration, exhibited lower MoCA scores compared to the PD group. This unexpected finding may be explained by the fact that patients with MSA exhibit more severe clinical symptoms and lower subjective cooperation ability. Additionally, the MoCA version we utilized is influenced by regional cultural background and population characteristics. Some items of the scale are challenging and the actual evaluation time is lengthy, which can pose difficulties for MSA patients and thus introduce bias in the scoring of the MoCA scale.

In conclusion, this study confirmed that CCM can serve as an objective and sensitive biomarker to differentiate PD and MSA. However, it is essential to note that our results require more extensive cohort studies, standardization of CCM techniques, and interpretation of the findings. Further longitudinal studies incorporating blood markers are needed to explore the diverse pathological mechanisms at the corneal level.

## Methods

### Subjects

PD (*n* = 78), MSA (*n* = 32), and healthy control subjects (HC) (*n* = 31) were recruited at the Department of Neurology, Affiliated Hospital of Xuzhou Medical University between October 2021 and October 2022. PD was diagnosed by the Queen Square Brain Bank criteria^30^. This study recruited patients diagnosed with possible and probable MSA based on the established diagnostic criteria^31^, and the diagnoses of these patients were also retrospectively confirmed after the publication of the latest diagnostic criteria^32^. Healthy individuals without any previous neurological disorders were chosen as controls, either from volunteers or spouses of patients. Study participants younger than 35 and older than 85 were excluded. The study was approved by the Ethics Committee of the Affiliated Hospital of Xuzhou Medical University. The study followed the principles of the Declaration of Helsinki for clinical investigations with human participants.

Concurrent chronic corneal pathologies, peripheral neuropathy caused by other known factors, chronic alcoholism, hepatic disease, active malignancy, refractive and cataract surgery history, and systemic diseases affecting the cornea like Sjogren’s disease, chronic kidney disease, and Fabry’s disease were among the exclusion criteria. Each participant provided written informed consent. Furthermore, none of the participants were treated with eye lenses or topical eye drops prior to the CCM examination. Movement disorders specialists reviewed each participant’s clinical profile carefully.

### Medical history and demographic characteristics

The participants’ sex, age, medical history, education level, and medications, including dopaminergic therapy, were documented. The calculation of disease duration is based on the date of diagnosis and the date of assessment. The blood tests (full blood count, glycosylated hemoglobin, B12, homocysteine, cholesterol) were performed to rule out other possible etiologies of neuropathy.

### Neurological assessment

In the ON state, section III (motor examination) of the International Parkinson and Movement Disorder Society/UPDRS (MDS-UPDRS) was used to assess motor severity^33^. Patients with MSA were assessed by the Unified Multiple System Atrophy Rating Scale I and II^34^ (UMSARS-I and UMSARS-II). Autonomic symptoms were evaluated by the Scale for Outcomes in Parkinson’s Disease for Autonomic Symptoms (SCOPA-AUT)^35^, which assesses the patient-reported manifestations across six dimensions of autonomic function: digestive, urinary, cardiovascular, pupillary, thermoregulatory, and sexual. Furthermore, the Orthostatic Grading Scale (OGS) was used to assess symptoms in patients with neurogenic orthostatic hypotension (NOH) and quantify the severity of OH symptoms^36^. The Non-Motor Symptoms Scale (NMSS) was used to assess the quality of life (QoL) and non-motor symptoms (NMS) of the patients. Cognitive function was assessed using the Chinese version (Beijing 7.1) of the Montreal Cognitive Assessment (MoCA) scale^37^. We used the Hoehn and Yahr scale to evaluate the disease stage.

### Ophthalmic assessment

Our optometrists performed all the ophthalmic assessments. We acquired Corneal confocal images using a laser scanning corneal confocal microscope: Heidelberg Retinal Tomograph III Rostock Cornea Module (HRT III RCM; Heidelberg Engineering GmbH, Heidelberg, Germany). The CCM images were quantitatively analyzed using the fully automated software ACCMetrics (M.A. Dabbah, Imaging Science, The University of Manchester, 2010)^38^, which can reduce observer-dependent bias. The current version of the software is optimized for CCM images captured by a Heidelberg HRT-III microscope, with 384 × 384 pixels and a field of view of 400 × 400 µm^2^ (resolution: 400/384 = 1.0417 µm). Using the Heidelberg microscope, CCM images can be automatically cleaned up by removing the information bars at the bottom.

The participant’s head and chin were stabilized using head/chin frames during the examination. The subject was given lidocaine to numb each eye and was told to focus on a white light with the opposite eye from the one being examined. During the examination, the charged couple device (CCD) camera was employed to accurately place the applanating cap on the cornea and track the camera’s precise position on the cornea’s surface. Multiple images of the subbasal plexus were taken and stored in a database, and 4 to 6 high-quality images from each eye were manually selected based on a quality measure by ophthalmology experts who were blinded to the patients’ diagnosis. Finally, approximately 2% of the area of the subbasal nerve plexus was selected and used for the quantification. Corneal nerve fiber density (CNFD): number of main nerve fibers per frame (no/mm^2^), corneal nerve branch density (CNBD): number of intersections between main nerves and secondary nerves per frame (no/mm^2^), corneal nerve fiber length (CNFL): the total length of all nerve fibers per frame (mm/mm^2^).

### Statistical analysis

The data analyses were performed using IBM SPSS version 26 and GraphPad Prism v. 8.02. The normality of the distribution was evaluated using the Shapiro-Wilk test. Numerical values are reported as the mean ± standard deviation (SD) for variables that adhere to a normal distribution. To compare multiple groups, an analysis of variance was conducted with Bonferroni as the post hoc test. The independent-sample t-test was employed to compare means of data that follow a normal distribution. The median (interquartile range) was used to express numbers, and the Mann-Whitney U test was employed for nonparametric data to assess variables that do not follow a normal distribution or exhibit non-homoscedasticity. Chi-square tests and Fisher’s exact tests were used to compare categorical variables. Partial correlation analysis was utilized to examine the correlation between CCM parameters and clinical measures within our cohort. To mitigate the influence of confounding factors, age, sex, and disease duration were selected as covariates for binary logistic regression. We performed three logistic regressions for each CCM metric (CNFD, CNBD, and CNFL are the dependent variables in each regression, respectively. Subsequently, receiver operating characteristic (ROC) curve analysis was conducted to assess the predictive capability of biomarkers or combination markers in diagnosing PD and MSA. Statistical significance was determined by a two-sided *p* < 0.05.

### Reporting summary

Further information on research design is available in the Nature Research Reporting Summary linked to this article.

### Supplementary information

Reporting Summary

## Data Availability

All data generated or analyzed during this study are included in this article. Further inquiries can be directed to the corresponding author.
